# Application of Recurrence Plot Analysis to Examine Dynamics of Biological Molecules on the Example of Aggregation of Seed Mucilage Components

**DOI:** 10.3390/e26050380

**Published:** 2024-04-29

**Authors:** Piotr Sionkowski, Natalia Kruszewska, Agnieszka Kreitschitz, Stanislav N. Gorb, Krzysztof Domino

**Affiliations:** 1 Institute of Theoretical and Applied Informatics, Polish Academy of Sciences, ul. Bałtycka 5, 44-100 Gliwice, Poland; piotr.sionkowski@gmail.com (P.S.); kdomino@iitis.pl (K.D.); 2 Group of Modeling of Physicochemical Processes, Faculty of Chemical Technology and Engineering, Bydgoszcz University of Science and Technology, 85-796 Bydgoszcz, Poland; 3 Department of Plant Developmental Biology, University of Wrocław, ul. Kanonia 6/8, 50-328 Wrocław, Poland; agnieszka.kreitschitz@uwr.edu.pl; 4 Department of Functional Morphology and Biomechanics, Kiel University, Am Botanischen Garten 1-9, D-24098 Kiel, Germany; sgorb@zoologie.uni-kiel.de

**Keywords:** time series analysis, recurrence plot, aggregation, molecular dynamics, seed mucilage, classical–quantum passage

## Abstract

The goal of the research is to describe the aggregation process inside the mucilage produced by plant seeds using molecular dynamics (MD) combined with time series algorithmic analysis based on the recurrence plots. The studied biological molecules model is seed mucilage composed of three main polysaccharides, i.e. pectins, hemicellulose, and cellulose. The modeling of biological molecules is based on the assumption that a classical–quantum passage underlies the aggregation process in the mucilage, resulting from non-covalent interactions, as they affect the macroscopic properties of the system. The applied recurrence plot approach is an important tool for time series analysis and data mining dedicated to analyzing time series data originating from complex, chaotic systems. In the current research, we demonstrated that advanced algorithmic analysis of seed mucilage data can reveal some features of the dynamics of the system, namely temperature-dependent regions with different dynamics of increments of a number of hydrogen bonds and regions of stable oscillation of increments of a number of hydrophobic–polar interactions. Henceforth, we pave the path for automatic data-mining methods for the analysis of biological molecules with the intermediate step of the application of recurrence plot analysis, as the generalization of recurrence plot applications to other (biological molecules) datasets is straightforward.

## 1. Introduction

Seeds and fruits of many plants produce a natural hydrogel substance—mucilage [1]. We can observe it in our daily life during food preparation of seeds such as flax, basil, chia, or plantain. Seeds of those plants, when we put them in the water, form gel-like, sticky, transparent capsules called mucilage envelopes [2]. Seed mucilage is a very soft material of great importance due to both its biological functions and promising applications in the design of new functional materials. Already utilized across various industries, such as food, pharmaceuticals, and cosmetics, its appeal lies in its non-toxic and biodegradable nature. It is also odorless, colorless, and tasteless. Some of its key benefits for industrial applications are its viscoelasticity and plasticity thanks to the distinct structural properties of its components and their interactions [3,4].

**Figure 1:**
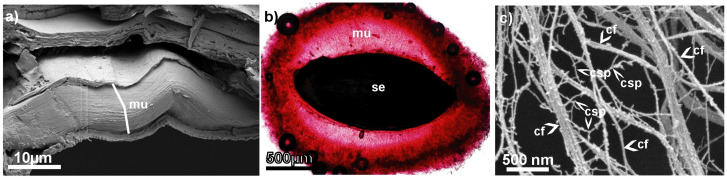
The seed mucilage structure before and after hydration with water. (**a**) Cross section of the mucilaginous cell showing pressed mucilaginous material in a dry state (mu) (SEM); (**b**) mucilage envelope (mu) formed after hydration around the seed (se) (here, mucilage stained with Ruthenium Red); (**c**) Critical point drying and subsequent SEM visualization of the spatial structure of the mucilage envelope. cf—cellulose fibrils; csp—cross-linking polysaccharides (pectins and hemicelluloses). For the details of the techniques, see [8].

Regarding its biological origin, mucilage represents a modified cell wall [5]. Every plant cell is surrounded by the cell wall, which determines the cell shape and protects the cell against mechanical or chemical damage and pathogens [6]. Typical, main components of the cell wall are polysaccharides, such as cellulose, hemicellulose, and pectins [7]. The same composition can be found in the mucilage envelope, but the polysaccharides occur here in different proportions. The dominating component of the mucilage can be pectins or hemicelluloses, which are responsible for water accumulation in the envelope. Cellulose can be an additional element of the mucilage and forms long fibrils that build a kind of scaffold for pectins and hemicelluloses. All the components form a net-like structure, where diverse interactions (ionic and hydrogen bonds and van der Waals forces) keep them together [8]. Mucilage is produced by mucilaginous cells building a single-layer seed coat on the seed surface. The mucilage material in the dry state is visible as a deposited, thick layer in the mucilaginous cells (cf., Figure 1a). The mucilage envelope rapidly forms after hydration (cf., Figure 1b) and can be divided into two main layers (based on the *Arabidopsis thaliana* model plant), namely (i) the outer layer, which is mainly built of unbranched rhamnogalacturonan I (RG1) pectin, which can be easily lost (washed out) from the mucilage and (ii) the inner layer, which composed of pectic polysaccharides like long, unbranched chains of homogalacturonan (HG), branched RG1, and cellulose fibrils [5,9].

Various biochemical studies revealed that hemicellulose chains (xylan) are linked to RG1 chains, facilitating the attachment of mucilage to cellulose microfibrils [3]. In the case of hemicellulose, xylan, xyloglucan, and arabinoxylan are often presented in plants with cellulose-based mucilage [10].

Using Critical Point Drying (CPD) and Scanning Electron Microscopy (SEM), the 3D net-like architecture of mucilage has been previously observed [8,10], namely the size, structure, and distribution of polysaccharides within the mucilage envelope (see Figure 1c). From CPD+SEM visualizations, the probable localization of specific mucilage components based on chain thickness and location has been deduced [8]. It remains challenging for experimentalists to precisely describe the intra- and intermolecular interactions within the entire network structure, which induce aggregation through non-covalent bonds such as hydrogen bonds (HBos) and hydrophobic–polar (HP) interactions. What is most important is that it is hard to observe the evolution of the network in various physicochemical conditions. Such information can be important, for example, in designing new materials according to the structure–property paradigm [11]. A recent publication [12] highlights the significance and ongoing relevance of this issue. The authors elucidated the stress–strain behavior of the plant epidermal cell wall based on a coarse-grained molecular dynamics model. This approach appears to be one of the most realistic models of the cell wall, as it encompasses the length scale necessary for investigating the origins of wall mechanics or cellulose-based networks. However, the use of coarse-grained models leads to the loss of certain system details, such as interactions with water, which is a crucial component in our study. There are also several studies describing interactions between cellulose and pectin or cellulose and hemicellulose as two-component systems [13,14,15,16,17]. However, there is still a need to explore the interactions within three-component systems simultaneously and examine the interaction preferences of the components. The models employed by experimentalists often tend to be overly simplistic and inadequately linked to quantum physical reality. Here, computer simulation methods such as molecular dynamics (MD) are helpful, as they can be supplemented by quantum mechanical methods.

Classical MD is a computer simulation technique in which the trajectories of atoms and molecules are established through numerical solutions of Newton’s equations of motion for a set of interacting particles. These forces, along with their potential energies, are frequently computed using interatomic potentials or molecular force fields (FFs). Also, novel neural networks and machine learning potentials have been developed recently [18,19,20]. In simulations of environments with a large number of atoms, classical mechanics force fields (FFs) such as AMBER or CHARMM are typically used. Even in these classical FFs, corrections to their parameters resulting from quantum mechanics calculations are introduced [21,22]. Namely, the corrections are based on obtained experimental or quantum mechanical data of small model systems [23] to improve parameters of the FF’s intermolecular terms, namely electrostatic forces and van der Waals interactions. The use of a parameter-rich classical FF allows for less computer-power use with the cost of loss of accuracy of the simulations. One should take into account that the quantum-based weakening or strengthening of the HBo is not considered in these classical FFs [24] (cf., Section 2.1). There are also quantum mechanics FFs, e.g., FFLUX [20], which is based on the quantum chemical topology approach, but such FFs are designed for small systems.

Another newer approach is ab initio molecular dynamics (AIMD) [23,25]. In this methodology, trajectories are generated by using forces computed directly from electronic structure (quantum mechanical) calculations just during the simulation process. In contrast to classical MD, this method allows for the breaking and formation of chemical bonds and considers electronic polarization effects. The AIMD method with a linear-scaling electronic structure theory overcomes the barrier of available computing power for such calculations because it scales very favorably to massively parallel computing systems and translates large, sparse matrix operations into highly parallel operations. Thus, strong GPUs allow this method to compute more than 100 million atoms (see, e.g., the CP2K simulation package [23]). It is worth mentioning that the FFLUX FF also uses AIMD results to compute many-body potential energy surfaces [20].

In the present study, the number of HBo and HP interactions was quantified for the model mucilage system undergoing an aggregation process at five temperatures ranging from 290 to 310 K. For such a relatively simple system, classical MD simulations with the AMBER FF (with the quantum corrections described above) were considered adequate, as we focused solely on standard, non-covalent interactions within the system immersed in water, which are well predicted by this semi-classical FF [22]. Time series data containing changes in intermolecular HBos; HBos between mucilage components, polysaccharides, and water (polysaccharides–water HBos; PW HBos); and intermolecular HP interactions as a function of time were obtained from these simulations and subsequently analyzed using the recurrence plot method.

We investigated the impact of small temperature changes on the aggregation of model mucilage components and assessed the utility of recurrence plot analysis for this purpose. The mentioned recurrence plot analysis is a dedicated tool used to analyze time series data from complex, chaotic systems [26]. A variety of automatic methods derived from recurrence plot analysis are available (see [27,28,29]); however, these methods are rather simple. The development of more advanced methods of data mining tied to recurrence plots is the new take from a data mining point of view. As such, recurrence plots have the potential to explore new methods for handling data from chaotic systems. We intend to pave the way for such an approach with the example of biological molecules data of plant seed mucilage. Thus, the main question addressed in this study is whether the assessment of recurrence plots can yield meaningful information on the rate of the aggregation process in biological systems and the stability of the network structure. This could have significant implications in the field of biology, as this information could provide insights into the mechanical properties of the structure.

## 2. Materials and Methods

The number of non-covalent interactions can be a quantitative measure of the aggregation of the macromolecules (polysaccharides) in the mucilage. Non-covalent interactions encompass various scales, from noble gas dimers to proteins (or other macromolecules) in aqueous solution and beyond. Diverse methods, such as first principles and force fields, have been successfully applied in this exploration. However, there is presently a lack of a fully integrated and consistent quantitative prediction method for non-covalent interactions across all system scales and research domains [20]. Please, keep in mind how important it is to consider the tight packing of biomolecules within living cells. For example, non-covalent interactions govern the entire structure and functioning of molecular machines. Such interactions aid also the matter self-organization process [30,31,32]. The structure of cellulose aggregates has also been theoretically revealed by the well-known fringed micelle model proposed by Herrmann et al. [33,34]. This model is consistent with [32], suggesting that cellulose molecules aggregate to form fringed micelles, which further aggregate to form larger, virtually fractal, and partially crystallized structures [35,36]. A quantum-based experimental method used for structure recognition in fringed-micelle polymers was reported in [37].

### 2.1. Physical Model with Reference to Quantum Mechanics

Since the present study was inspired by [38], a brief description of aggregation in biological systems as a passage between quantum and classical mechanics is introduced in this section, building upon the groundwork presented in the Introduction.

Scientists studying mucilage emphasize that the nature of interactions among its components is still not fully explored [3,10]. Nevertheless, the importance of intermolecular non-covalent bonds, such as ionic or HBos, is often emphasized, as they influence many physicochemical and rheological properties of the mucilage [10,39]. Both types of interaction strictly result from quantum mechanics. In the first type, an electron is transferred from one atom to another, thus achieving a more energetically favorable state. This typically occurs when one atom has a significantly higher electron affinity, while the other has a much lower ionization energy (the energy required to remove an electron) [40]. However, HBos classically treated as interactions dominated by electrostatic forces are modified by quantum effects, which diminish weak HBos and strengthen relatively strong ones. This arises from a competition between anharmonic intermolecular bond bending and intramolecular bond stretching [24,41]. In various studies, the importance of HBos has been overstated due to their weak and environmentally sensitive nature [42]. Moreover, intra- or intermolecular HBos always have to compete with HBos originating from water. Thus, it should be noted that in addition to HBos, there is another important (and stronger) type of interaction present within such mucilage, namely hydrophobic–polar (HP) interactions [42]. The importance of the hydrophobic effect in cellulose-based mucilage has been highlighted, for example, by Lindman, who has stated that this is a major reason why cellulose is resistant to most solvents [43]. Whether HP interactions can also be explained by quantum mechanics was studied in [44], where hydrophobicity was investigated at the molecular level. The authors deduced that solute and water molecules tend to cluster independently rather than together. These intermolecular associations represent a substantial portion of the enthalpic contribution to phase separation, which reveals the relevance of solute–solute interactions as hydrophobic.

All those non-covalent bonds support adhesion, which is crucial for mucilage functions, such as enabling seeds to attach to various natural surfaces (e.g., soil), preventing seed dispersal or, on the contrary, to attach to animals (e.g., birds), allowing for seed dispersal [3,10,45]. Another key factor underpinning the distinct rheological properties of mucilage is side-chain distribution in hemicelluloses [39] and pectins and the degree of esterification of pectins [46,47]. Other important factors include the pH of the solvent and the content of cross-linking agents, such as calcium ions, which (quantum-based) interact with pectins [46], as well as with cellulose [48]. To be more specific, pectin molecules contain carboxylic acid groups, which can undergo ionization depending on the pH of the surrounding environment. Decreasing the pH results in a reduction in electrostatic repulsion among the pectin chains, which primarily facilitates chain association via hydrogen bonding [49]. Additionally, carboxyl groups in pectins can form bonds with Ca^2+^ ions, and the number of these groups depends on the degree of methylation of pectin [47]. Ionic bridges between pectins can also make the entangled chain network stronger [47].

Accordingly, a classical–quantum passage arises from the scenario depicted above, wherein the quantum nature of the interactions strongly influences the structure and properties of meso- or even macroscopic objects, such as mucilage. This passage has also been observed in many other biological systems, e.g., in [50], where quantum mechanics calculations incorporating solvent effects successfully identified the preferred molecular structure of topotecan, an anticancer drug, in solution at various pH levels. Another interesting example of this phenomenon was studied by Wybranowski et al. [51], who studied the determination of the affinity of drugs (warfarin and flurbiprofen) for human serum albumin (the most abundant protein in blood plasma) through fluorescence anisotropy measurements. In this example, the (quantum-based) interactions between the drug and protein play a crucial role in transporting bound drugs to the tissues. More extensive discourse on the classical–quantum crossover is available in [52]. A problem of the boundary between classical stochastic and a quantum stochastic description within a mesoscopic matter-aggregating system was addressed by Gadomski and Kruszewska [53], employing Nelson’s quantum–stochastic approach [54].

### 2.2. Molecular Dynamics Simulation

**Figure 2:**
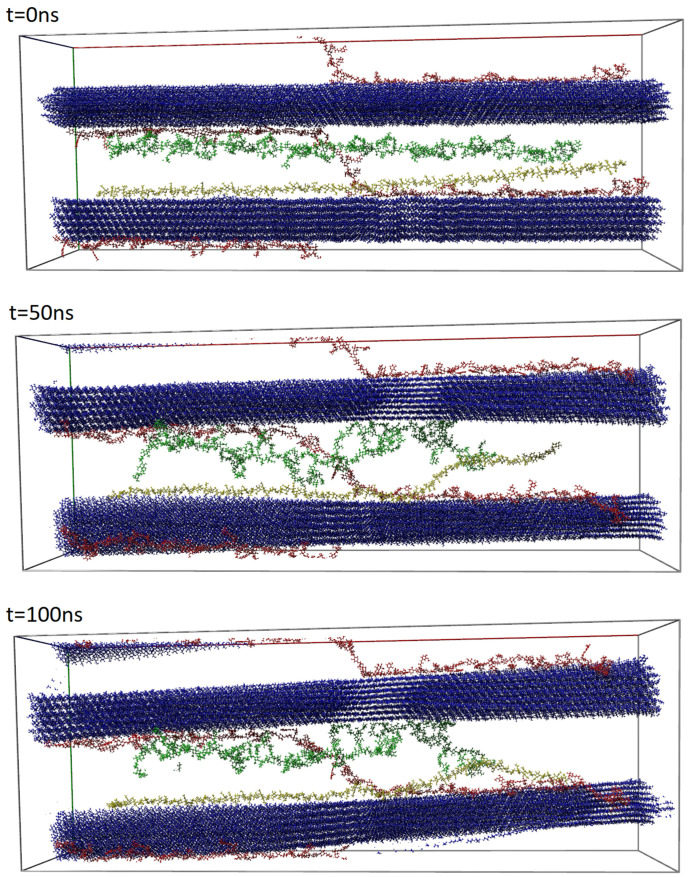
Structures of a cellulose, hemicellulose, and pectins complex in explicit water solution at a temperature equal to 300 K (solution is transparent in the image). Components are colored as follows: celluloses, blue; hemicelluloses, red; RG1 pectin, green; HG pectin, yellow. Snapshots come from YASARA and show three stages of simulations: at t=0 ns (upper), at t=50 ns (middle), and at t=100 ns (bottom) of MD simulations [70].

We performed all-atom classical MD simulations of the model system consisting of two cellulose fibrils, two chains of xylan (hemicellulose), one RG1, and one HG chain (two different pectins) in an explicit water solution (see Figure 2). All these components are representative here for only one seed mucilage type—cellulose mucilage [2]. Scenes and molecule simulations were prepared and performed using YASARA software (version 22.8). The system components’ lengths were chosen specifically to give them a chance to build a network of non-covalent bonds. Such an entangled system allowed us to observe interactions between all three polysaccharide types at once.

The main components of the model system are the cellulose fibrils—bundles of 36 linear polymer microfibrils, each with a degree of polymerization of 40. They were created by Cellulose Builder software [55], which is based on the model proposed by Ding and Himmel [55,56]. The chemical structure of xylan was obtained from PubChem (ID 129539666); modified to obtain chains with a degree of polymerization equal to 128; and branched with arabinose, ferulic acid, and glucuronic acid according to Oliveira et al. [57]. In the case of pectins, CHARMM-GUI software (version 3.8) [58,59] was used to generate pectin chains (HG and RG1) with a backbone length of 80 saccharides, as described in [60]. For HG, the backbone was modified by 32 methyl esterifications at the C-6 carboxyl position (low-methyl esterified HGs) and by 32 O-acetylations at the O-2 or O-3 position [60,61]. In this study, an RG1 model similar to the one presented in Figure 3.19 of [62] was used. Its backbone had 24 branches, namely eight arabinofuranoses, eight galactoses, and eight mixes of these two.

After necessary minimization of the model system to remove clashes, the simulation was run for 100 ns using the AMBER14 force field [22], which uses parameters appropriate for most known molecules (e.g., GLYCAM06 [63] for carbohydrates), and TIP3P for water. The van der Waals force cut-off distance was 10 Å [64]. The particle mesh Ewald algorithm was used to compute long-range interactions (e.g., electrostatic interactions) [65]. Simulations were performed under the following conditions: five temperatures of 290, 295, 300, 305, and 310 K, and a pressure of 1 atm (NPT ensemble) [66]. The complex was immersed in aqueous solutions with pH = 7.4. Periodic boundary conditions were applied to a box with dimensions roughly equal to 431×158×69 Å^3^. The number of atoms in the model system was about 500,000 (ca. 120,000 H_2_O molecules). A Berendsen barostat and thermostat were used to maintain constant pressure and temperature, respectively (relaxation time of 1 fs) [67]. The equations of motion were integrated with multiple time steps of 1.25 fs for bonded interactions and 2.5 fs for non-bonded interactions. In the considered simulations, the time step between saved states of the system equaled 100 ps. Thus, the time series for 100 ns of simulations corresponded to 1000 save points. Snapshots of the model system before, in the middle, and after 100 ns of MD simulation are presented in Figure 2. All simulations were duplicated with different seeds of a random number generator to verify whether similar and reproducible results were obtained (results are presented in Supplementary Materials Figures S1–S3).

The numbers of HBos and HP interactions were calculated using the standard algorithms for the MD simulations described previously in [68,69] and in the YASARA manual.

### 2.3. Recurrence Plot Method

For recurrence plot analysis, we selected datasets from simulations of molecular dynamics increments of the number of intermolecular HBos, HBos between mucilage components and water (PW BHos), and intermolecular HP interactions (taken as ΔN/Δt to obtain a non-monotonic function, where *N* is the number of specific interactions). Through this method, we aimed to observe alterations in the dynamics of aggregation in the model mucilage. This analysis was performed by an in-house-written data processing program in Python 3.9, a developed version of the one used in [71]. Together with systematic description, we illustrate the recurrence plot procedure on the toy example of the following artificial input vector:

(1) $$\vec{x}=\left(1,\frac{1}{2},1,\frac{1}{2},1\right).$$

Recurrence plot analysis can be summarized by the following algorithm.

#### 2.3.1. Input

The input is an *L*-long univariate (equally time-spaced) time series ($\vec{x}$) with elements ($x_{i}$) that, in our case, represent increments of the number of intermolecular bonds/interac- tions at given timestamps represented by *i*s.

#### 2.3.2. Proceeding

1. **Data compression**. In the first step, we perform a compression of time series into the following sub-series:
  (2) $${\vec{y}}_{i}^{\left(d,\tau \right)}=\left(x_{i},x_{i+\tau },x_{i+2\tau },\ldots ,x_{i+d\tau }\right),$$
  i.e., by putting $x_{i}$ at the beginning, spacing by τ, and keeping $x_{i+d\tau }$ at the end. Such series (of length d+1) are numbered by *i*, where i∈{1,2,…L−dτ} (${\vec{y}}_{i=1}^{\left(d,\tau \right)}$) corresponds to the initial part of $\vec{x}$, while ${\vec{y}}_{i=L-d\tau }^{\left(d,\tau \right)}$ corresponds to the rear part of $\vec{x}$, etc.
  Henceforth, the procedure is parameterized as follows:
  - parameter τ—delay time;
  - parameter *d*—embedding dimension of the system.
  In our approach, the particular value of each of these parameters was computed by algorithmic methods discussed in [71], namely time delay was computed by the *first non-significant auto-correlation* introduced therein, and dimensional embedding was computed by the Cao method [72]. If we compare more time series, majority voting among data inputs is performed to determine one value of each parameter for a fair comparison. Generally, the recurrence plot method is dedicated to data originating from a chaotic physical system. Hence, subsequent elements of the data series are supposed to carry similar information. Besides, after a long enough evolution, a chaotic system is supposed to return to the neighborhood of the starting point, and the corresponding data record is supposed to reflect repeating information.
  Referring to the toy example of the artificial input vector in Equation (1) and assuming τ=2 and d=1 (and L=5), we have L−dτ=3 in the following sub-series:
  (3) $$\begin{array}{cc} {\vec{y}}_{1}^{\left(d,\tau \right)} & =\left(x_{1},x_{3}\right)=\left(1,1\right)\\ {\vec{y}}_{2}^{\left(d,\tau \right)} & =\left(x_{2},x_{4}\right)=\left(1/2,1/2\right)\\ {\vec{y}}_{3}^{\left(d,\tau \right)} & =\left(x_{3},x_{5}\right)=\left(1,1\right) \end{array}.$$
2. **Sub-series transformation**. From L−dτ sub-series (${\vec{y}}_{i}^{\left(d,\tau \right)}$), one creates a (L−dτ)×(L−dτ) matrix (M(λ)) with zero and ones entries as follows:
  (4) $$m_{i,i^{\prime }}\left(\lambda \right)=\left\{\begin{array}{cc} 0 & \;\mathrm{if}\;∥{\vec{y}}_{i}^{\left(d,\tau \right)}-{\vec{y}}_{i^{\prime }}^{\left(d,\tau \right)}∥\leq \lambda \\ 1 & \;\mathrm{elsewhere}\; \end{array}\right.$$
  The idea beyond this representation relies on the comparison of subsequently compressed series (${\vec{y}}_{i}^{\left(d,\tau \right)}$ and ${\vec{y}}_{i^{\prime }}^{\left(d,\tau \right)}$) [73] with the norm $∥{\vec{y}}_{i}^{\left(d,\tau \right)}-{\vec{y}}_{i^{\prime }}^{\left(d,\tau \right)}∥$. Ideally, such a distance should be smaller than the threshold value (λ) determining the accuracy of compression in the previous step. In practice, the particular value of $∥{\vec{y}}_{i}^{\left(d,\tau \right)}-{\vec{y}}_{i^{\prime }}^{\left(d,\tau \right)}∥$ reveals the local dynamics of data. In detail, maps as in Equation (4) display interesting patterns that can be analyzed later on by sophisticated methods (see [74]). The fraction of zeros in M(λ) from Equation (4) is the *recurrence rate* in the literature, and there is no single broadly accepted method concerning its determination.
  Referring to the toy example in Equations (1)–(3), we present two examples of an $M\left(\lambda \right)\in {\left\{0,1\right\}}^{3\times 3}$ matrix, given the following two distinct λ parameters:
  (5) $$M\left(\lambda =0.1\right)=\begin{array}{cc} \begin{array}{ccc} i\prime =1 & 2 & 3 \end{array}\\ \left(\begin{array}{ccc} 0\;\; & 1 & 0\\ 1\;\; & 0 & 1\\ 0\;\; & 1 & 0 \end{array}\right) & \begin{array}{c} i=1\\ 2\\ 3 \end{array} \end{array}\;M\left(\lambda =0.9\right)=\begin{array}{cc} \begin{array}{ccc} 1 & 2 & 3 \end{array}\\ \left(\begin{array}{ccc} 0 & 0 & 0\\ 0 & 0 & 0\\ 0 & 0 & 0 \end{array}\right) & \begin{array}{c} 1\\ 2\\ 3 \end{array} \end{array}$$
  Recall, that the *recurrence rate* is $\frac{5}{9}$ in the λ=0.1 case and 1 in the λ=0.9 case.
3. **Recurrence plot creation**. A recurrence plot is created by turning the zeros and ones in Equation (4) into white and black pixels (dots). The recurrence plot naturally visualizes data in a time window with a length of *d* and spacing of τ. Such recurrence plots can be analyzed either manually (the state-of-the-art approach) or in an automatic manner (a new approach if advanced tools of image processing are applied). Here, we are left with the open question as to what particular information is tied to the recurrence plot and how it depends on parameters (and *the recurrence rate* in particular).
4. **Entropy**. To assess information tied to the recurrence plots, we first use the most straightforward approach, namely the entropy approach as proposed in [27] and by us in [71]. Following this, we compute the Shannon entropy from the distribution of specific features of the recurrence plot. Following [27], we estimate topological entropy, which measures the total complexity of the orbit structure of the chaotic system, as the Shannon entropy of the distribution of parallel-to-diagonal lines. In detail, a histogram is made for the lengths of zero sequences along the diagonal direction. Normalizing the histogram and thinking of it as a probability distribution ($p_{j}$), a Shannon entropy can be derived through the following equation:
  (6) $$S=-\sum_{j}\left(p_{j}\log p_{j}\right),$$
  where $p_{j}$ is the probability of a sequence of length *j* and the summation is over all sequence lengths. Then, we analyze such entropy for the entire range of the *recurrence rate* (from 0 to 1) to assess whether the information tied to the recurrence plot varies with the *recurrence rate* and select its most suitable value.
  Concerning the toy example in Equations (1), (3), and (5),
  - For λ=0.1, we have j∈{1}, $p_{1}=1$, and S=0; and
  - For λ=0.9, we have j∈{1,2}, $p_{1}=p_{2}=1/2$, and S=0.693.
5. **Recurrence plot analysis and automatic pattern recognition**. If we refer in more detail to the recurrence plot layout, we can conclude that in regions close to its diagonal (*i* close to $i^{\prime }$ in Equation (4)) sub-series from close time instants are analyzed, while in the regions away from the diagonal (*i* is far from $i^{\prime }$), series from distinct time instants are analyzed. Furthermore, if we observe the dark cross hitting the diagonal at certain subsequent sub-series (i.e., $i\in I_{\mathrm{cross}}$), the dynamics are tied to ${\vec{y}}_{i}^{\left(d,\tau \right)}$ such that $i\in I_{\mathrm{cross}}$ should differ from the dynamics of the rest of the data series. Such $I_{\mathrm{cross}}$ is interesting from the point of view of pattern recognition and data analysis. To search for $I_{\mathrm{cross}}$ automatically, we introduce the following cross-detection procedure:
  (a) Compute the column-wise weight of the recurrence plot in Equation (4) ($w_{i}=\sum_{i^{\prime }}m_{i,i^{\prime }}$), and create weight vector $\vec{w}$;
  (b) Smooth $\vec{w}$ to ${\vec{w}}_{\mathrm{SMA}(k)}$ by applying the simple moving average (SMA) of size *k*, where *k* is the parameter;
  (c) Normalize ${\vec{w}}_{\mathrm{SMA}(k)}$ to ${\tilde{\vec{w}}}_{\mathrm{SMA}(k)}$ by subtracting the mean and dividing by the standard deviation;
  (d) Determine the *k* parameter for SMA to maximize the highest value of the normalized vector, namely
    (7) $$k=\mathrm{argmax}(\max{\tilde{\vec{w}}}_{\mathrm{SMA}(k)});$$
  (e) Save a *k* value equal to the width of dark crosses;
  (f) Determine cross centers as local maxima of the smoothed $SMA_{k}\left(\vec{w}\right)$ that are greater than the threshold standard score (number of standard deviations).
  Converting $SMA_{k}\left(\vec{w}\right)$ into a standard score enables us to work with a vector of unitless, standardized values. This facilitates comparison across different datasets and enhances the method’s versatility. With standard scores established, it becomes feasible to suggest a default threshold value for cross-detection, such as 2σ or 3σ (σ denotes standard deviation). An alternative solution would require the inspection of each dataset individually and would be more challenging to automate. Please, keep in mind that this method can be easily generalized for white cross-detection.

#### 2.3.3. Output

The output is a recurrence plot with detected patterns (crosses).

Concerning the toy example in Equations (1), (3), and (5), for λ=0.1, we expect a dark cross with a width of 1, hitting the diagonal at i=2. From this cross analysis, the sub-series starting at i=2, namely ${\vec{y}}_{2}^{\left(d,\tau \right)}=\left(x_{2},x_{4}\right)=\left(1/2,1/2\right)$, is expected to differ from the other sub-series, namely ${\vec{y}}_{1}^{\left(d,\tau \right)}=\left(x_{1},x_{3}\right)=\left(1,1\right)$ and ${\vec{y}}_{3}^{\left(d,\tau \right)}=\left(x_{3},x_{5}\right)=\left(1,1\right)$.

## 3. Results and Discussion

Examples of simulation snapshots for a system at a temperature of 300 K are presented in Figure 2. When comparing the systems before and after simulation, one can observe a twist in both celluloses and a change in their orientations. This ability of cellulose to twist has been observed very rarely in plants, e.g., in the cell wall of green alga *Micrasterias denticulate* [75]. Twisting of microfibrils was observed in different model simulations of cellulose in the presence of water and was attributed to trans-glycosidic linkages due to hydrogen bonds [76,77]. The ability to twist can have an important effect, e.g., on the structural and mechanical properties of diverse polymers including cellulose [76].

Nevertheless, the positions of the celluloses relative to each other remain stable due to the two hemicellulose chains, which, attached to both celluloses, create a kind of scaffold. Many authors have postulated the strengthening role of xylan in the cell wall due to the interaction with cellulose fibrils [78,79,80]. Xylan also plays an important role in maintaining the cellulose architecture in the mucilage envelope [81]. In studies on the seed mucilageof model plant *Arabidopsis thaliana*, it has also been suggested that xylan may link RG1 to cellulose fibrils [3]. Many mucilage polysaccharides demonstrate the presence of side chains. Due to this property, it is also possible to create diverse interactions between mucilage polysaccharides. This is presumably important for keeping this special (spatial) structure of mucilage, for maintaining the mucilage at the seed surface, and for water accumulation [5,8,81].

Initially arranged parallel to the celluloses and placed near them, hemicelluloses aggregated via non-covalent bonds, namely HBos and HP interactions. In this stage of preliminary studies, there were no ionic interactions in the system, as it was immersed in water without the addition of any ions. Investigating a system with various added ions will be the subject of further study.

**Figure 3:**
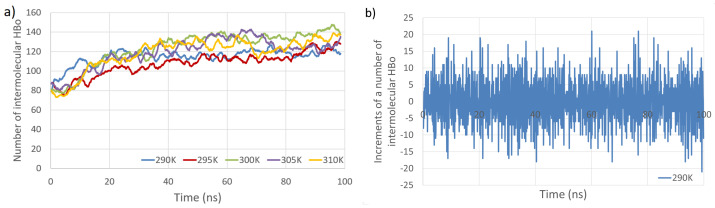
Total number of intermolecular HBos as a function of simulation time at five temperatures (**a**). Example of analyzed time series (**b**). The time series consists of increments in the number of intermolecular HBos at a temperature of 290 K (computed from the blue line in (**a**)).

**Figure 4:**
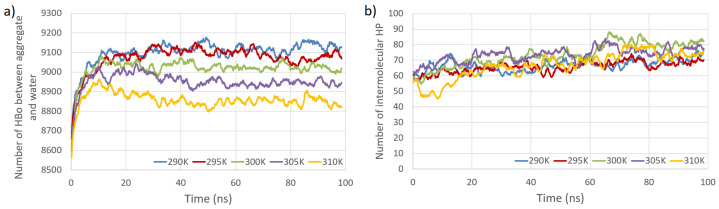
Total number of HBos between model polysaccharides and water molecules (PW HBo) as a function of simulation time at five temperatures (**a**). Total number of intermolecular HP interactions as a function of simulation time at five temperatures (**b**).

The time evolution of the number of intermolecular HBos and the changes in this number at subsequent time points (time series) are presented in Figure 3. Figure 4 depicts the time evolution of the number of intermolecular HP interactions, as well as the number of PW HBos.

The primary observations from Figure 3 and Figure 4 indicate that the temperature dependence of the number of intermolecular HP interactions is negligible, and a very small dependence on the number of intermolecular HBos is observed. Nevertheless, the temperature dependence of HP interactions. without a doubt, does exist, as described in [82], where it was demonstrated that the temperature dependency of the Gibbs free energy of hydrophobic and directly hydrogen-bonded solutes was opposite to that of bridged hydrophilic solutes. Therefore, a possible explanation for the temperature independence observed in our HP interactions (at least in the small temperature range tested) could be attributed to the presence of a large molecular system with a multitude of diverse hydrophobic and polar interactions (celluloses and pectins are amphiphilic, so have some hydrophobic regions and some hydrophilic ones), resulting in various local dependencies on temperature, both positive and negative, which may cancel each other out. However, a discernible dependence is noticed in the case of PW HBos. The reason for this is that at higher temperatures, water molecules possess greater thermal energy, and water has a lower density. These factors affect the mobility of water molecules, facilitating the detachment of hydrogen atoms from water molecules bound to polysaccharide atoms, thereby enabling their return to the bulk solution. This, in turn, liberates space for HBos originating from the polysaccharides. Figure 3b illustrates the chaotic, noise-like fluctuations in the number of intermolecular HBos at 290 K. Comparable patterns are observed in the plots for the remaining interactions; therefore, only one example is presented here. The potential inclusion of information within this noise can be explored through recurrence plot analysis.

**Figure 5:**
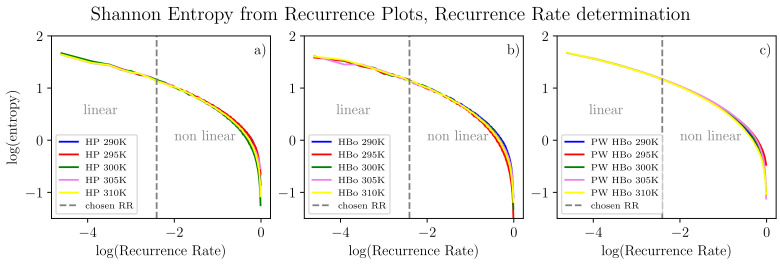
Entropy computed from recurrence plots according to point 4 in Section 2.3 for various temperatures and inter-molecular bonds: HP (**a**), HBo (**b**), PW HBo (**c**). A linear relation between the logarithm of entropy and the logarithm of the *recurrence rate* can be observed to the left of the dashed line. The following parameters were used for all data series (see Equation (2)): τ=2 and d=1. Determination of these parameters was performed by the automatic method described in point 1 in Section 2.3 for each data series, then by majority voting.

From all input data in the form of Equation (2), we algorithmically computed the delay time (τ) and embedding dimension (*d*) as discussed in Section 2.3. For a fair comparison, single values of these parameters (d=1 and τ=2) were selected by majority voting among all datasets. Henceforth, each series can be compressed to many two-point sub-series (see Equation (2), where d=1), each still carrying meaningful information about the dynamics of the system. Given values of the *d* and τ parameters, for sound analysis of recurrence plots, the *recurrence rate* parameter (the same best value for all data series) also has to be determined. The theoretical evaluation of the recurrence threshold is tied to the size of the attractor of the chaotic system under investigation. Here, we are motivated by [83], according to which one expects the *recurrence rate* to be in the region where scaling between log(λ) (see Equation (4)) and the logarithm of the *recurrence rate* is still linear. To extend this approach, we examined analogous scaling but with reference to Shannon entropy computed for the recurrence plot [27], which is meant to assess information tied to the orbit structure and its size in particular. Referring to Figure 5, this entropy decreases monotonically with the *recurrence rate*. This is most probably due to the observation that the higher the *recurrence rate*, the less sensitive the plot is to small details of the dynamics and the more it reflects the average behavior of the system. In this sense, we are moving from a regime of detailed analysis to a regime of averaged analysis of the system. In our approach, we intend to assess both regimes. Henceforth, we select a *recurrence rate* on the edge between linear and non-linear relation. Such an approach is expected to assess the phase transition-like behavior and, hence, interesting features of the system. In the current approach, we selected this point manually, but algorithmic generalization is possible. In detail, algorithmic detection of such a point would involve testing linear vs. non-linear models of data sub-series in observation windows, searching for the changing point between these two models and finally applying majority voting among datasets. Alternatively, one could use a single line fit that minimizes the quadratic error of the fit towards all the curves in scope.

Selected entropy values (computed from the recurrence plot of the *recurrence rate* selected in Figure 5) in comparison with a number of particular bonds are presented in Table 1. Bear in mind that in the HBo case, we have an order of magnitude fewer bonds (approximately a hundred) than in the PW HBo case (approx 9 thousand). In the HP interaction case, there is an even smaller number of interactions (half of that for HBos). The entropy value was highest for the the PW HBo case, which was expected, as this one is the largest and most chaotic system. As mentioned before, each of the analyzed data series was compressed to two-point sub-series (see Equation (2), where d=1). The stability of the compression can be deduced from the stable (between time series) plots of information entropy presented in Figure 5. The above observation is positive from the point of view of data compression.

**Figure 6:**
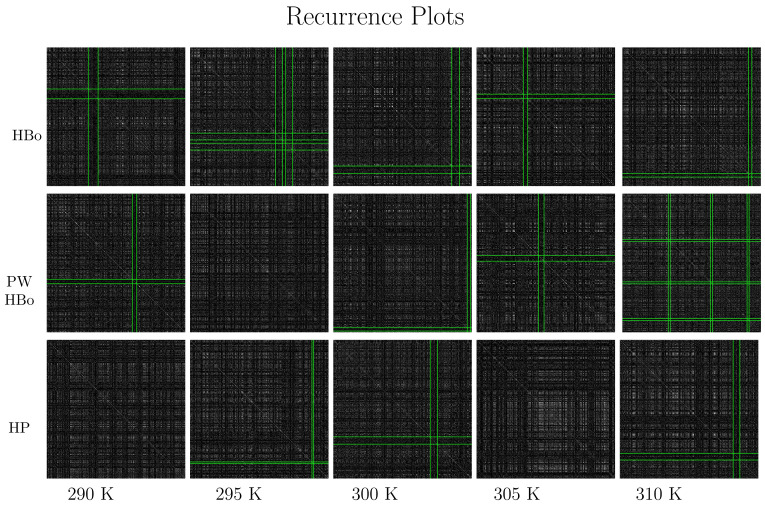
Recurrence plots and cross detection: intermolecular HBo (upper panel), PW HBo (middle panel), and intermolecular HP interaction (lower panel). Dark crosses detected by the algorithms are bounded by green lines. Such dark crosses are meant to indicate simulation times (starting from the upper left of the plot and following the diagonal downward) where dynamics are expected to differ from the average. Notice the temperature-dependent pattern of such regions for HBos and random behavior for other interactions. Additionally, notice the white regions in the case of HP contacts; these also carry meaningful information about the system dynamics and, in particular, may be associated with some form of oscillations. Parameters of the recurrence rate were determined by majority voting as follows: τ=2, d=1, and RR=9%; the cross was detected with a threshold standard score equal to 2.5 σ (standard deviations) (see the algorithm in Section 2.3).

Recurrence plots are presented in Figure 6, which shoes the dark crosses detected by the algorithm described in Section 2.3. In the HBo case (top panel), the cross moves toward the bottom-left corner with the increase in temperature, except for at 305 K. Such a cross (while crossing the diagonal) is expected to mark the time range where the system’s dynamics differ from the general dynamics, and the system may be in some sort of transition. The 305 K case does not fit this pattern, suggesting some specific dynamics at this temperature. This indicates the necessity of a deeper analysis of this case, focusing on factors such as the degree of cellulose surface solvation or aggregation within the rest of the components. These factors could potentially influence both intra- and intermolecular interaction strengths across various temperatures [84]. For PW HBos, the cross locations look more random, which can be tied to the high noise level of such a system; nevertheless, the dynamics are similar. A deeper analysis of Figure 6 also emphasizes other (less significant) dark crosses extended over the full length of the graph. Therefore, the entire time series may display some form of anomalous dynamics. The presence of such dark crosses may be a manifestation of the local change of the phase space [27]. This may suggest that the phase space is expanding in those regions, and there is almost no return to the original state of simulations. We observed somewhat similar but more uniform patterns in the total van der Waals energy data, namely data with meaningful random factors in their dynamics (see Figures 6–9 in [71]). The discussed patterns were similar in different simulation runs with different seeds of the random generator, as presented in Supplementary Materials (cf., Figure S3).

Concerning the lower panel of Figure 6, white regions can be observed, especially for higher temperatures. This is also a meaningful observation tied to molecular dynamics. In particular, if there is a recurrence plot of increments in a series of white regions separated by dark ones, we may expect a stable oscillation (of a number of HP bonds in our case) around the mean value. This interesting pattern was expected, as HP bonds are stronger than the HBos, although they are fewer in number (cf., Table 1).

## 4. Conclusions

We have qualitatively analyzed the concept resembling fringed-micelle involvement in the mucilage [35,36]. For the advanced analysis of the dynamics of the aggregation process, we applied algorithmically created recurrence plots. Broadly speaking, these images can be analyzed either manually (the state-of-the-art approach in the literature) or automatically. Referring to the first approach, we observed two structures, namely dark crosses and white squares. The former correspond to a time sub-sequence in which the dynamics of the system differ from the average dynamics. The latter (white squares near the diagonal in particular) correspond to the local stabilization of increments and were observed for hydrophobic–polar interactions at higher temperatures. This coincides with the observation that the temperature dependence of hydrophobic–polar interactions does exist (in analogy to our temperature range), as described in [82], where it was demonstrated that the temperature dependency of hydrophobic and directly hydrogen-bonded solutes was opposite to that of bridged hydrophilic solutes.

Referring to the second approach, we proposed a simple automatic method for dark cross detection, which points out the direction for automatic methods in the analysis of recurrence plots. Inspired by this, more general automatic methods can be evolved. Through this approach, we discovered that the number of HBos exhibited some unconventional dynamics over the course of the simulation, which are temperature-dependent. Specifically, as the temperature increases, the occurrence of these unusual regions is delayed. This suggests that the system undergoes a slight change in its state within the time range indicated by these dark crosses, becoming more aggregated and stable thereafter.

One line of future research will refer to the application of image segmentation methods [85] and object detection (see recent methods proposed in [86,87]) with reference to the AI approach. An example of classification of time series (EEG in particular) via recurrence plots classified by deep artificial neural networks was described in [88]. Analogous methods can be potentially applied for the detection of particular patterns in recurrence plots.

## Figures and Tables

**Table 1:** The system characteristics for selected temperatures: 290 K (left) and 310 K (right). The highest entropy is observed for the PW HBo case, which is meant to be the most chaotic because of the high noise level included in those data. Furthermore, in the case of a PW HBo, entropy does not depend on temperature meaningfully.

Interaction Type	Entropy	n.o. Bonds		Entropy	n.o. Bonds	
		**Mean**	** σ **		**Mean**	** σ **
Intermolecular HBo	3.15	114.8	9.9	3.08	118.7	17.4
PW HBo	3.20	9098.4	84.0	3.20	8855.3	69.4
Intermolecular HP	3.17	66.7	6.8	3.12	66.7	10.2

## Data Availability

Data and processing codes will be made available by the authors subject to any reasonable demand.

## Supplementary Materials

The following supporting information can be downloaded at: https://www.mdpi.com/article/10.3390/e26050380/s1, Figure S1: Number of all intermolecular HBos as a function of simulation time in five temperatures (a). Example of analyzed time series (b). The time series consists of increments in the number of intermolecular HBos at temperature 290K (computed from the blue line from picture (a)); Figure S2: Number of all HBos between model polysaccharides and water molecules (PW HBo) as a function of simulation time in five temperatures (a). Number of all intermolecular HP interactions as a function of simulation time in five temperatures (b); Figure S3: Recurrence plots for data with other initial seed, cross detection: intermolecular HBo (upper panel), PW HBo (middle panel), and intermolecular HP (lower panel). Temperatures from left (290K, 295K, 300K, 305K, 310K). By majority voting among the whole data set, we used constant τ=2 and d=1 and RR=9%. The cross was detected with the threshold standard score equal to 2.5σ (standard deviations).

## Abbreviations

The following abbreviations are used in this manuscript:

FF	Force field
HBo	Bydrogen bond
PW HBo	Polysaccharides–water hydrogen bond
HP	Hydrophobic–polar
MD	Molecular dynamics
AIMD	Ab initio molecular dynamics
PDB	Protein Data Bank
HG	Homogalacturonan
RG1	Rhamnogalacturonan I
