# Supplementary material for: Application of Recurrence Plot Analysis to Examine Dynamics of Biological Molecules on the Example of Aggregation of Seed Mucilage Components
Source: Entropy (Basel). 2024 Apr 29;26(5):380. doi: 10.3390/e26050380 (PMC11119629; doi:10.3390/e26050380)
Supplement: Supplementary file 1 [file entropy-26-00380-s001.zip › entropy-2957042-supplementary/sup_mat/Supplem_Recurrence_plot_analysis_for_molecular_non_covalent_interactions_in_seed_mucus.pdf]

Article

Supplementary materials to: Application of recurrence plot analysis to examine dynamics of biological molecules on the example of aggregation of seed mucilage components.

Piotr Sionkowski [1], Natalia Kruszewska [2], Agnieszka Kreitschitz [3], Stanislav N. Gorb [4], Krzysztof Domino [1]

[1] Institute of Theoretical and Applied Informatics, Polish Academy of Sciences, ul. Bałtycka 5, 44-100 Gliwice, Poland; piotr.sionkowski@gmail.com, kdomino@iitis.pl;
[2] Group of Modeling of Physicochemical Processes, Faculty of Chemical Technology and Engineering, Bydgoszcz University of Science and Technology, 85-796 Bydgoszcz, Poland; nkruszewska@pbs.edu.pl;
[3] Department of Plant Developmental Biology, University of Wrocław, ul. Kanonia 6/8, 50-328, Wrocław, Poland; agnieszka.kreitschitz@uwr.edu.pl;
[4] Department of Functional Morphology and Biomechanics, Kiel University, Am Botanischen Garten 1-9, D-24098 Kiel, Germany; sgorb@zoologie.uni-kiel.de;
* Correspondence: nkruszewska@pbs.edu.pl

In the supplementary materials, we present additional results from computer experiments, specifically focusing on the number of intermolecular interactions (Figures 1 and 2) and recurrence plots (Figure 3) from the second experiment iteration of aggregation process in the model mucus. These data are akin to those shown in Figure 5 of the main body of the article but are derived from a distinct series of simulations initiated with a different seed for the random number generator.

Referring to Figure 3, we can observe similar locations of crosses detected for the case of HBo than in the other series of data discussed in the main body of the article. This is the argument for the robustness of the method. For PW HBo and HP cases, we observe random locations of crosses, which coincides again with the data series analyzed in the main body of the article. Finally, as for the case of HP, fewer white regions are observed than in the main body of the article. However in analyses here series, we observe (especially for 310K) many randomly located crosses, what is the other (than white regions observed in the main body of the article) evidence of oscillations. (White regions separated by dark regions are the full evidence of oscillations).

Citation: . *Entropy* **2024**, *0*, 0.
https://doi.org/

Received:
Accepted:
Published:

Publisher's Note: MDPI stays neutral with regard to jurisdictional claims in published maps and institutional affiliations.

Copyright: © 2024 by the authors. Submitted to *Entropy* for possible open access publication under the terms and conditions of the Creative Commons Attri- bution (CC BY) license (https://creativecommons.org/licenses/by/4.0/).

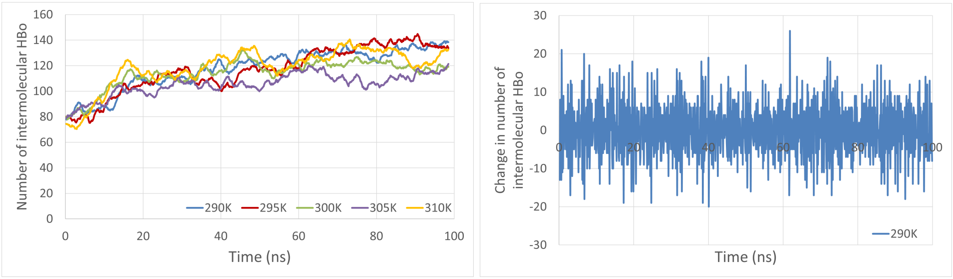

Figure 1. Number of all intermolecular HBos as a function of simulation time in five temperatures (a). Example of analyzed time series (b). The time series consists of increments in the number of intermolecular HBos at temperature 290K (computed from the blue line from picture (a)).

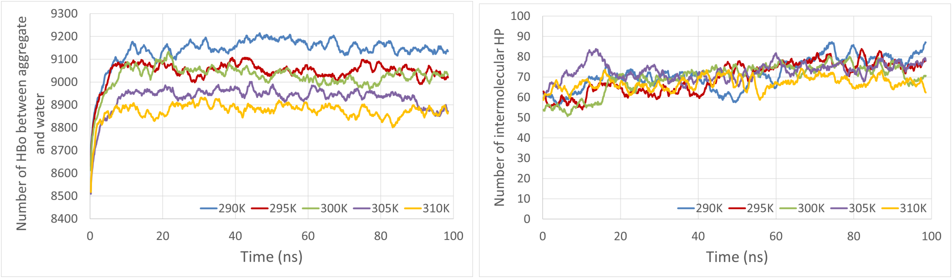

Figure 2. Number of all HBos between model polysaccharides and water molecules (PW HBo) as a function of simulation time in five temperatures (a). Number of all intermolecular HP interactions as a function of simulation time in five temperatures (b).

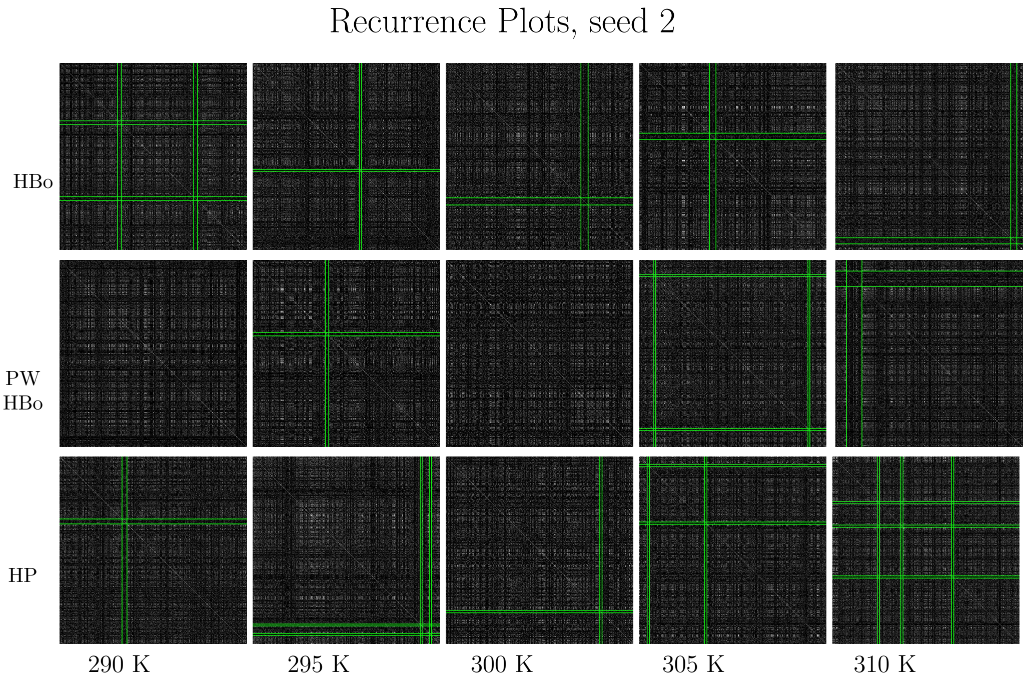

Figure 3. Recurrence plots for data with other initial seed, cross detection: intermolecular HBo (upper panel), PW HBo (middle panel), and intermolecular HP (lower panel). Temperatures from left (290K, 295K, 300K, 305K, 310K). By majority voting among the whole data set, we used constant $\tau = 2$ and $d = 1$ and $RR = 9\%$. The cross was detected with the threshold standard score equal to 2.5σ (standard deviations).
