# Supplementary figures and images for: Application of Recurrence Plot Analysis to Examine Dynamics of Biological Molecules on the Example of Aggregation of Seed Mucilage Components
Source: Entropy (Basel). 2024 Apr 29;26(5):380. doi: 10.3390/e26050380 (PMC11119629; doi:10.3390/e26050380)

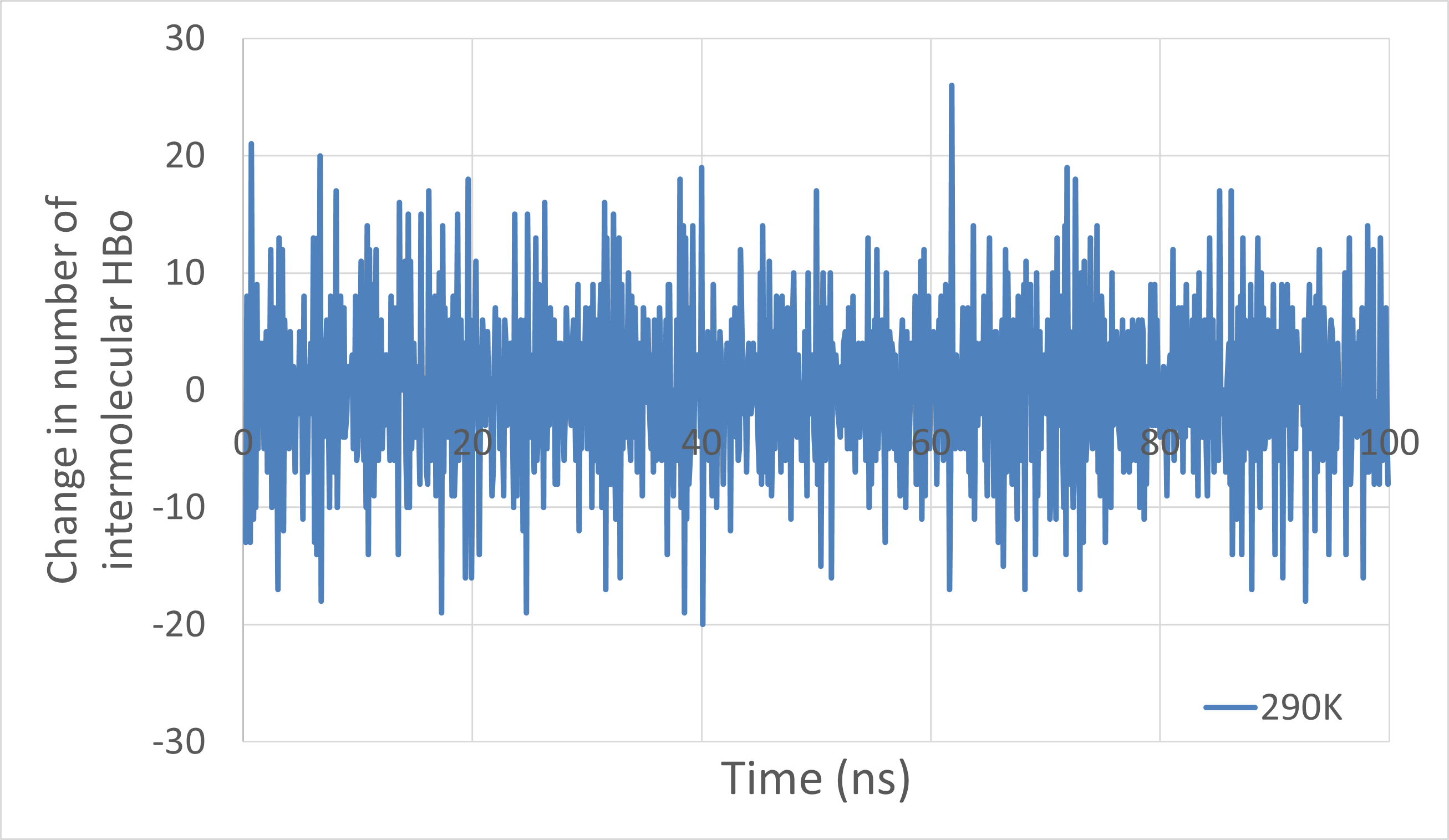

Supplement: Supplementary file 1 [file entropy-26-00380-s001.zip › entropy-2957042-supplementary/sup_mat/pics/supmat_pics/cel6_s2_deltaHBo.png]

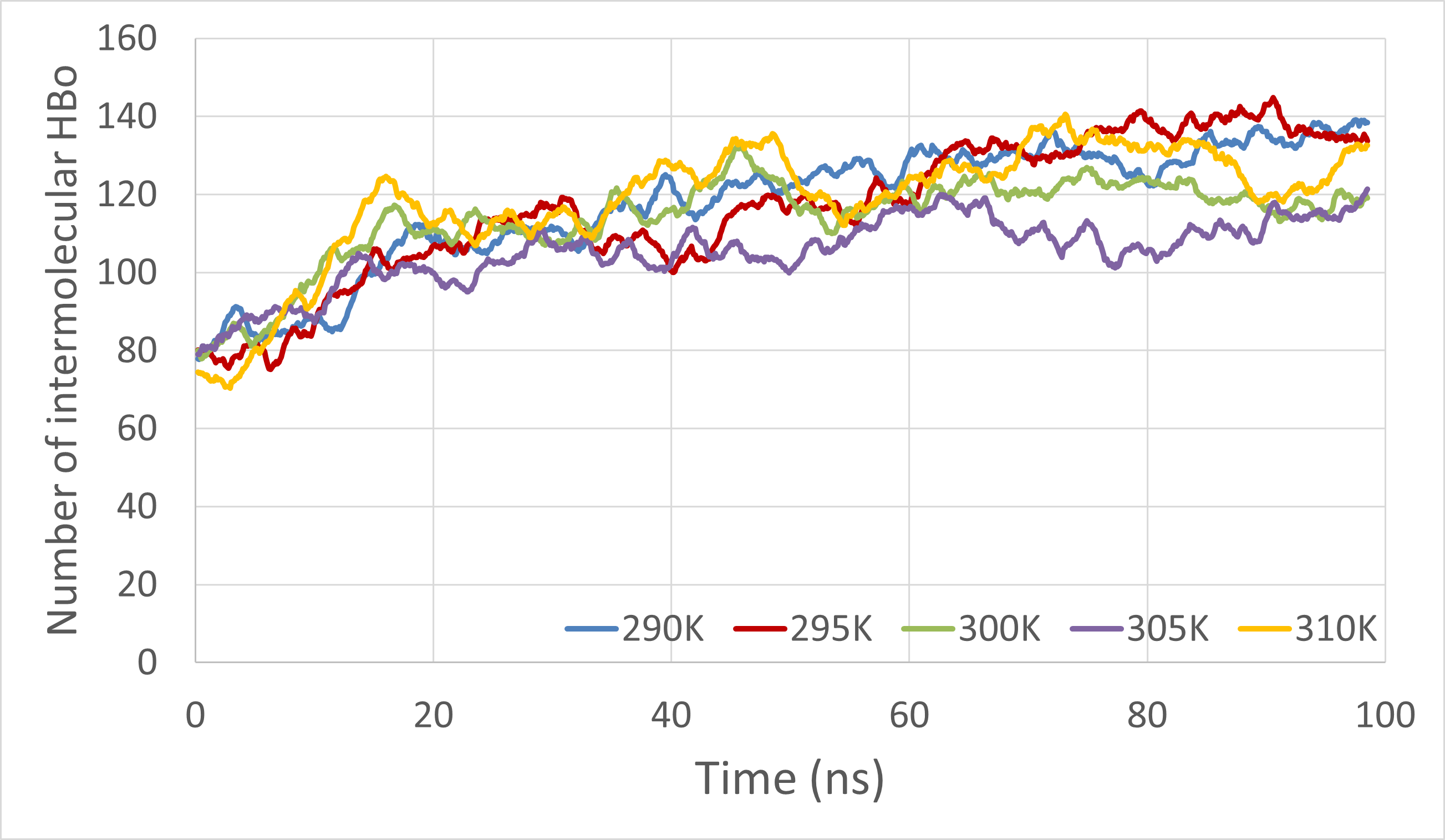

Supplement: Supplementary file 1 [file entropy-26-00380-s001.zip › entropy-2957042-supplementary/sup_mat/pics/supmat_pics/cel6_s2_HBo.png]

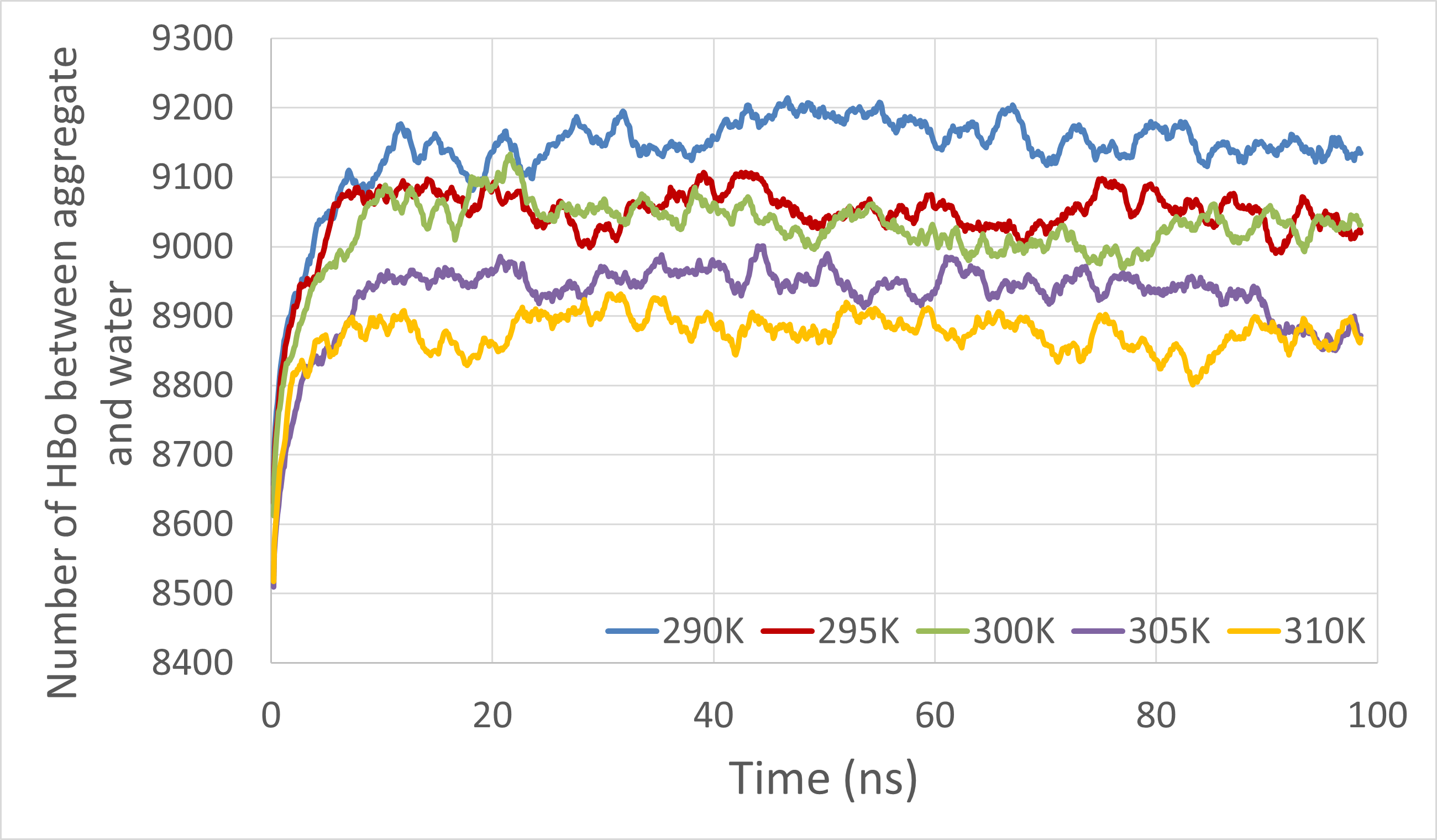

Supplement: Supplementary file 1 [file entropy-26-00380-s001.zip › entropy-2957042-supplementary/sup_mat/pics/supmat_pics/cel6_s2_HBoH2O.png]

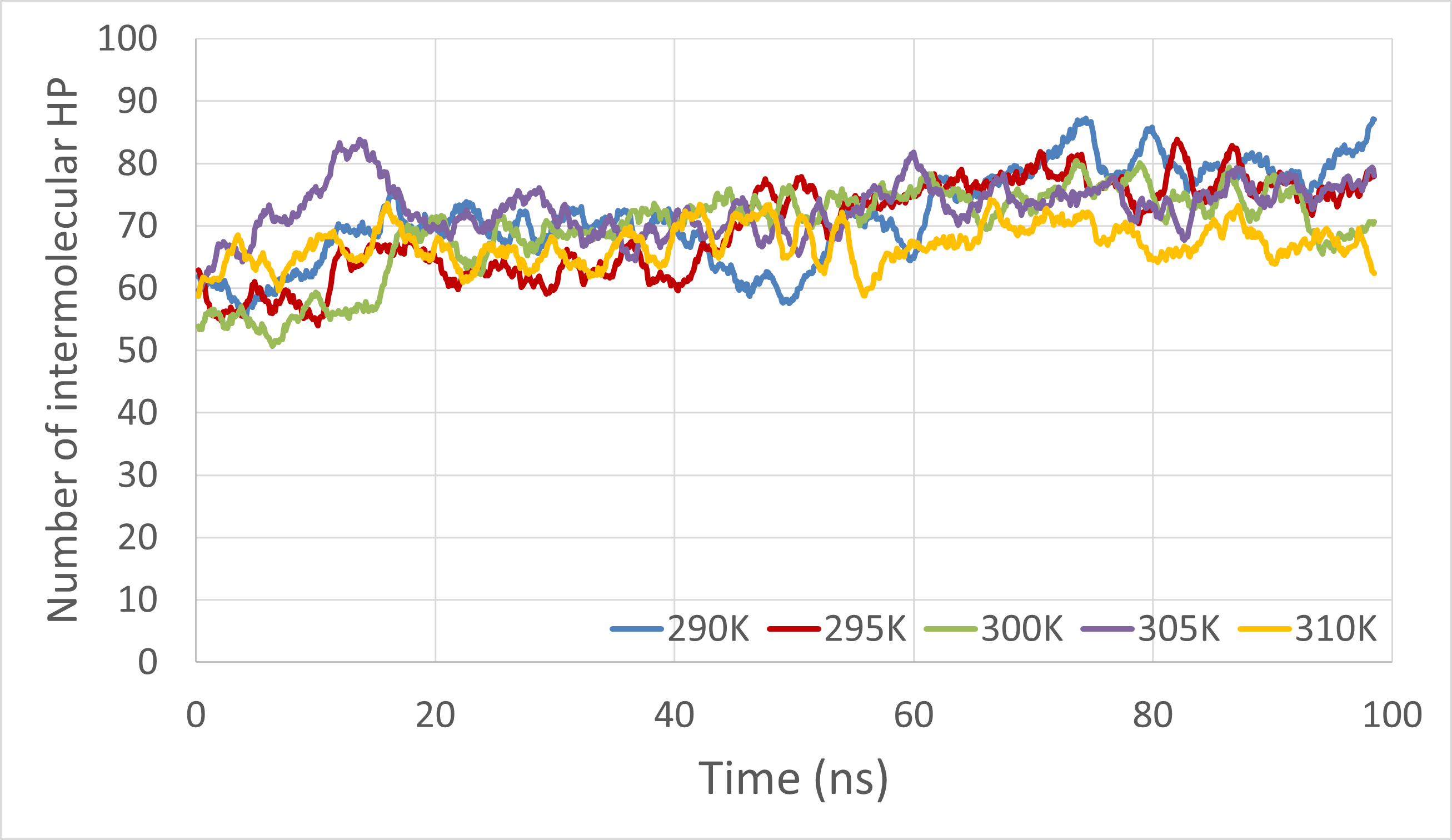

Supplement: Supplementary file 1 [file entropy-26-00380-s001.zip › entropy-2957042-supplementary/sup_mat/pics/supmat_pics/cel6_s2_HP.png]

# Recurrence Plots, seed 2

HBo

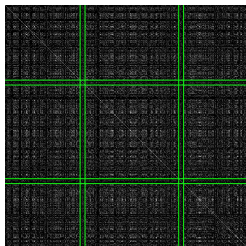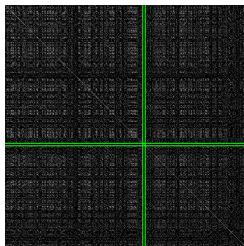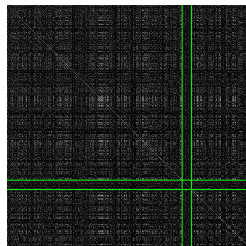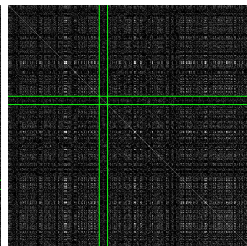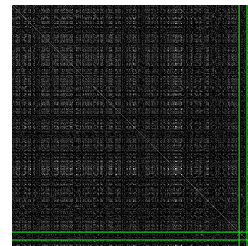

PW  
HBo

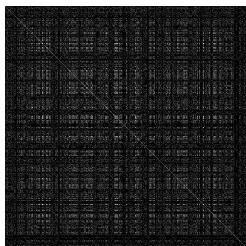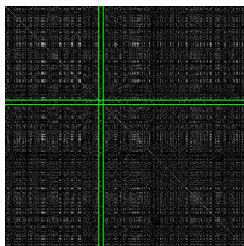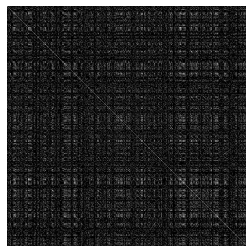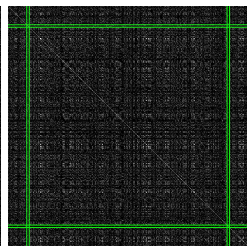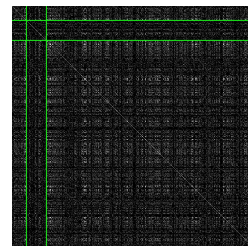

HP

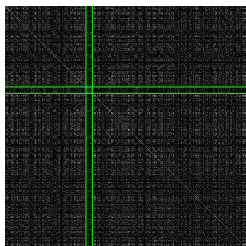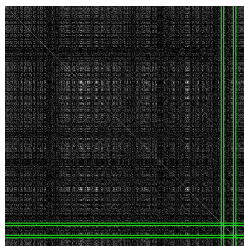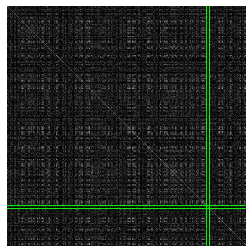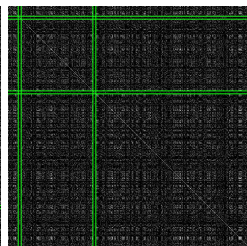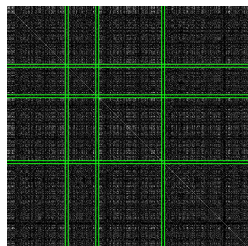

290 K

295 K

300 K

305 K

310 K

Supplement: Supplementary file 1 [file entropy-26-00380-s001.zip › entropy-2957042-supplementary/sup_mat/pics/supmat_pics/Reccurence_plot2.pdf]
